# PET and the multitracer concept in the study of neurodegenerative diseases

**DOI:** 10.1590/1980-57642015DN94000343

**Published:** 2015

**Authors:** Henry Engler, Andres Damian, Cecilia Bentancourt

**Affiliations:** 1 MD. PhD - Uruguayan Centre of Molecular Imaging (CUDIM), Montevideo, Uruguay.; 2 MD - Uruguayan Centre of Molecular Imaging (CUDIM), Montevideo, Uruguay.

**Keywords:** PET, neurodegeneration, F-flourodeoxyglucose, PIB, Alzheimer's disease, Creutzfeldt-Jakob disease, PET, neurodegenerativas, F-flourodeoxyglucose, PIB, Doença de Alzheimer, Doença de Creutzfeldt-Jakob

## Abstract

The complexity of the pathological reactions of the brain to an aggression caused by an internal or external noxa represents a challenge for molecular imaging. Positron emission tomography (PET) can indicate *in vivo,* anatomopathological changes involved in the development of different clinical symptoms in patients with neurodegenerative disorders. PET and the multitracer concept can provide information from different systems in the brain tissue building an image of the whole disease. We present here the combination of ^18^F-flourodeoxyglucose (FDG) and N-[^11^C-methyl]-L-deuterodeprenyl (DED), FDG and N-[^11^C-methyl] 2-(4'-methylaminophenyl)-6-hydroxybenzothiazole (PIB), PIB and L-[^11^C]-3'4-Dihydrophenylalanine (DOPA) and finally PIB and [^15^O]H2O.

## Abstract (pt)

A complexidade das reações patológicas do cérebro à agressões causadas por noxa interna ou externa representa um desafio para a imagem molecular. Tomografia por emissão de positron (PET) pode indicar, *in vivo,* alterações anatomopatológicas envolvidas no desenvolvimento de diferentes sintomas clínicos em pacientes com desordens neurodegenerativas. PET e o conceito de multitraçador pode fornecer informações de diferentes sistemas no tecido cerebral, construindo assim uma imagem da doença como um todo. Nós apresentamos neste artigo a combinação de ^18^F-flourodeoxyglucose (FDG) e N-[^11^C-methyl]-L-deuterodeprenyl (DED), FDG e N-[^11^C-methyl] 2-(4'-methylaminophenyl)-6-hydroxybenzothiazole (PIB), PIB e L-[^11^C]-3'4-Dihydrophenylalanine (DOPA) e finalmente, PIB e [^15^O]H2O .

## INTRODUCTION

The study of neurodegenerative diseases has experienced a qualitative leap since the introduction of molecular imaging of pathological processes *in vivo*. PET tracers have the potential to reveal changes in the different stages of many diseases in the brain. They improve the diagnoses increasing our understanding of the pathological processes in the central nervous system.

Different tracer combinations can be the way to characterize brain diseases with a higher level of accuracy. The multitracer concept can help us to achieve a more accurate classification of brain diseases and open the way for better therapeutic strategies.

In this paper we highlight the use of the multitracer concept for the characterization of neurodegenerative diseases and its implications in patient management. The Table 1 describes the different tracers we are going to cover in this review.

**Table 1:** PET molecular probes and their clinical use.

Radiotracer	Uptake mechanism	Clinical use
^18^F-flourodeoxyglucose (FDG)	Uptake by glucose transporters and trapped into the cell after phosphorylation by the enzyme hexoquinase	Alzheimer's disease, frontotemporal degeneration, Creutzfeldt Jakob disease, lymbic encephalitis, etc.
[^11^C-methyl] 2-(4'-methylaminophenyl)- 6-hydroxybenzothiazole (PIB)	Binding to amyloid-β peptide	Alzheimer's disease, amylodosis
L-[^11^C]-3'4-Dihydrophenylalanine (DOPA)	Measure of the DOPA- decarboxylase activity at the level of presynaptic terminals. Evaluation of presynaptic integrity	Parkinson's disease, atypical parkinsonisms, endocrine tumours
N-[^11^C-methyl]-L-deuterodeprenyl (DED)	Monoaminooxidase-B (MAO-B) inhibitor. Uptake by reactive astrocytes.	Creutzfeldt Jakob disease, Alzheimer's disease
[^15^O]H_2_O	Gold standard for non invasive measurement of cerebral blood flow (CBF)	Alzheimer's disease, frontotemporal degeneration, epilepsy, arterio-venous malformations

## FDG AND DED

^18^F-flourodeoxyglucose (FDG) is actively transported into the cell by a group of glucose transport proteins (GLUT) and phosphorylated. FDG is trapped in the cells as FDG-6-Phosphate avoiding the glycolysis and giving an indirect measure of the glucose uptake in the tissues.

N-[11C-methyl]-L-deuterodeprenyl (DED) binds irreversibly to the enzyme Mono-Amino-oxydase -B (MAO-B), which is expressed by reactive astrocytes and can be used as a marker for reactive astrocytosis.^1-8^

The combination of DED, indicating reactive astrocytosis and FDG, showing changes in the neuronal function, allows us to differentiate between diseases with simultaneous astrocytosis and neuronal death from diseases with astrocytosis and concomitant increased glucose uptake indicating inflammation.

We have used this combination of tracers to differentiate Creutzfeldt-Jakob disease (CJD) from other degenerative processes. CJD is a prion disease clinically characterized by sudden dementia, ataxia and myoclonia. The anatomopathological hallmarks in the brain include: spongiform changes, astrocytosis and neuronal death.

Today there is no cure for CJD, but some other diseases can be treated and therefore it is crucial to achieve an early diagnosis. Inflammation can be produced by an exogenous (bacteria, virus, etc.) or an endogenous agent (autoimmune diseases or paraneoplastic limbic encephalitis)^9^ that is caused by antineuronal antibodies produced in reaction to body neoplasms. In these patients the clinical symptomatollogy can appear as dementia of rapid onset. Sometimes the laboratory tests can help in the diagnosis, but in other cases it is not possible to differentiate between these clinical entities with this information.

The different patterns obtained with PET and appropriated tracer combinations may allow a differential diagnosis modifying the treatment. It is important not only to exclude CJD but also to suggest other diagnoses.

**Figure 1:**
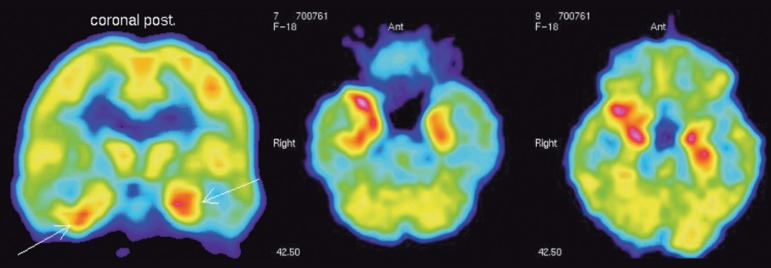
Patient with suspect CJD. The patient, however, had paraneoplasic limbic encephalitis. Left, coronal view: high FDG uptake bilaterally in the medial temporalcortex. Center and right: transaxial view. The FDG pattern is different to that seen in confirmed cases of CJD.

In one patient with dementia of rapid onset and symptoms similar to those found in CJD, high FDG uptake was observed in the limbic system (Figure 1). The medial temporal lobes including the amygdala, the hippocampus and the parahippocampus were the most affected regions.^2^

This pattern of uptake was very different to the pattern described with FDG in patients with confirmed CJD.^2^ Paraneoplastic limbic encephalitis was suspected, a whole body FDG PET was performed and a tumour was found in the left lung.

An autopsy of the patient revealed an adenocarcinoma of the lung and tumoural antibodies against the brain were demonstrated. The PET examination could quickly distinguish this patient from the other patients with CJD.

In this patient, the quantitative FDG examination proved to be sufficient to make the differentiation between these clinical entities.

Other patient with suspected CJD had a glucose uptake pattern similar to that found in Alzheimer's disease (AD). The metabolism was bilaterally decreased in the temporoparietal regions, but it was conserved in the central parts of the brain and the occipital cortex.

The FDG examination indicating hypometabolism in the parietotemporal areas with conserved metabolism in basal ganglia, cerebellum and sensory motor cortex suggested the possibility that the patient had AD instead of CJD, but further investigations (biopsy of a salivary gland) revealed

Sjögren's disease. Treatment with corticoids had a dramatic effect in the patient's symptoms.

Parieto-occipital hypometabolism is a conspicuous finding observed mainly in MRI-negative neuropsychiatric systemic lupus erythematosus (SLE). As this cerebral region is located at the boundary of the blood supply territories of all three major arteries, it could be the most vulnerable zone of the cerebrum and may be affected at early stages of the cerebrovascular disease.^10^

An autoimmune disease, producing inflammatory meningo-encephalitis or a perivascular inflammation that affect this region might create a pattern of glucose uptake which is not possible to differentiate with FDG from AD. Patients with Sjörgen's disease can present with symptoms of AD.^11^

It is important to differentiate between these diseases because a dementia with an onset corresponding to CJD or AD may be caused by immunological mechanisms: (System Lupus Erithematosus (SLE), Sjögren disease or a paraneoplasic phenomenon) and therefore can be treated with specific drugs. The result, particularly in the case of Sjögren's disease or SLE, can be dramatic, reversing a dementia condition in hours by administration of corticoids and cyclophosphamide.^11^ Even dementias produced by antineuronal tumour antibodies can be reversed by the extirpation of the tumour.^12^ In a patient with CJD we demonstrate the congruence between pathological findings and PET results, as expressed by the combination DED/FDG.^13^

In the case of rapid onset dementias, a brain investigation with FDG could be followed by a scan of the whole body to quickly discard the possibility of an unknown primary tumour producing antineuronal antibodies. If a brain scan shows hypermetabolism and the body scan is negative, autoimmune diseases or infectious diseases may be suspected. If the brain scan indicates asymmetric hypometabolism, the examination with DED can reveal high ratio DED/FDG suggesting CJD. Hypometabolism similar to that found in AD, but negative to PIB retention, may be caused by a disease affecting the vascular system.

## FDG AND PIB

The combination of FDG and PIB has helped us to understand better the dynamic process in the *in vivo* amyloid formation. The deterioration in cognition seems related to the decrease in the brain's glucose metabolism, but not directly to the increase in amyloid depositions.

The accumulation of amyloid in the brain appears to be an early process in the development of AD that increases to a certain level (possibly many years before the debut of the symptoms) and then reaching an equilibrium between aggregation and degradation.^14^

The glucose uptake decreases slightly in the beginning of the disease, reaching a critical point in which it becomes pronounced enough to be detected by PET FDG.

Because the metabolic changes occur later in the development of the disease, FDG can be used to confirm a diagnosis when the symptoms indicate dementia. The tracer however, is not able to discriminate between healthy persons and AD patients in the early stage of the disease in asymptomatic individuals, whereas PIB offers the possibility to detect amyloid depositions when the symptoms are not evident.

FDG in combination with PIB increases the possibility to perform differential diagnoses. If the PIB examination is negative and FDG reveals a pattern similar to that found in AD, it is possible that a disease with vascular anatomopathological substratum (autoimmune disease?) underlies the symptoms.

Several questions arise from the evidences presented above.

**1) Is the presence of amyloid in the brain a process that can be related to aging without consequences for normal functions?** It have been suggested a common origin in the pathologic processes that lead to neurofibrillary tangles and amyloid plaques in both aging and AD [15]. In a study performed in 1999, neurofibrillary tangles were found in the brain of all 39 nondemented persons examined, especially in the hippocampal and parahippocampal areas. The average tangle concentration was found to increase exponentially with age. In contrast, plaques were absent in some of the patient brains up to 88 years of age.

Other nondemented patients presented with widely distributed neuritic as well as diffuse plaques throughout the neocortex and limbic structures. It has been proposed that they represent "preclinical" AD.^16^

We suggest that the presence of amyloid is always a sign of degeneration and the depositions will disturb and injure the brain, causing dementia.

Like a city in which the recollection of refuse has been stopped and the garbage is blocking the streets preventing cars from circulating, the amyloid depositions and neurofibrillary tangles block interneuronal communication.

The brain has a highly developed capacity to compensate damage produced by the presence of strange substances accumulating in cytoplasm and interstitium and perturbing functions.

This capacity to compensate is not exclusive of the brain. All the organs in the human body have a reserve that allows normal functions when the tissue is affected by a disease.

We have seen in PET clinical routine extensive tumours invading a whole brain hemisphere before they cause symptoms.

Symptoms of Parkinson's disease (PD) appear when the degeneration of the presynaptic pathway has reached 30-50% of the striatonigral component. The diagnosis with PET can often be made because the damage is extended enough. We suggest that the brain compensates the slow and progressive biochemical and mechanic damage originated by the increasing presence of amyloid depositions and neurofibrillary tangles until a critical point when the collapse of the function is a fact without the possibility of return.

Although some studies have confirmed that asymptomatic patients may present positive PIB scans, there is a general agreement in the idea that PIB is very sensitive and has a high negative predictive value in the detection of AD. A negative PIB scan would be very important to discard AD and suggesting other possible diagnoses.^17-19^

If a person having amyloid depositions could live long enough, she or he should develop symptoms of AD. This hypothesis is supported by recent metaanalysis indicating that with longer follow-up periods, the accuracy of PIB to predict AD conversion in Mild Cognitive Impairment (MCI) patients improves significatively.^20^

Education, and aspects of occupational experience have been indicated as factors that may delay the clinical manifestations of AD.^21^ Other researchers suggest that the effect of education is modest.^22^ The mechanism underlying such a delay could be the presence in educated or trained people of more "activated" neurons from the "reserve pool", which could supply the effects of neuronal death.

**2) Not all patients fulfilling international criteria for AD show the presence of amyloid depositions.** A discussion concerning new classifications of dementia diseases seems necessary. Frontotemporal dementia (FTD) is a syndrome incuding many entities with different anatomopathological background.

We suggest that the AD diagnosis must be changed to "Alzheimer's syndrome" or "Cognitive Deficit of Alzheimer's type" (CDAT) because there are many diseases expressing the same symptoms and producing similar changes in glucose uptake.

The diagnosis of AD must be established in patients with "Alzheimer's syndrome" who have positive PET examinations with PIB or other well-proved amyloid markers. The absence of amyloid depositions is the most questioning result to the diagnosis of AD.

We have examined patients with "Alzheimer's syndrome" without amyloid depositions and it is possible for these patients to have, for example, a disease with a different pathological basis (vasculitis?), which is necessary to investigate.

Since physicians do not treat only symptoms, we need to clarify the underlying pathology to find the proper treatment for patients expressing similar clinical symptoms.

MCI is considered a transitional stage between normal aging and dementia, especially in early AD. It is known that MCI may have multiple causes, including AD and other forms of dementia, as well as depression and various psychiatric disorders. Because MCI is a frequent syndrome, there is a need to establish new methods for predicting the progression to AD.

In a previous study^23^ we demonstrate that PIB retention in MCI patients is an intermediate step between healthy controls (HCs) and AD patients. There are MCI patients who can have either high or low PIB retention. Eleven of twenty-one MCI patients showed high PIB retention in the frontal, parietal and temporal cortices.

The seven MCI patients that at follow-up converted to AD showed significant high levels of amyloid retention in the brain when compared to MCI non-converters and HCs. These seven converting MCI patients significantly differed as a group from the non-converts regarding a higher proportion of ApoE-4 carriers, impairment in episodic memory, lower cerebral spinal fluid (CSF) A-beta 1-42 and higher PIB retention. In general, we did not detect in this study decreased glucose metabolism in cortical brain areas as earlier reported in MCI patients.^24,25^ Nevertheless, in some individual MCI patients, decreased regional cerebral metabolic rate of glucose consumption (rCMRglc) was found in such areas as the parietal and temporal cortices. The non-converters in the MCI group had significant higher glucose uptake than AD patients whereas for the converters the difference was less.

In clinical routine we have observed increased glucose uptake in some of the patients with the diagnosis MCI.^26,27^ The first reaction of neurons against an unspecific injury is a hypermetabolic response. Injured neurons increase the glucose uptake by an enhanced glycogenesis, possibly as a form to economize energy necessary to repair the damages.

Hypermetabolism in patients with MCI may indicate some inflammatory process affecting the brain. These patients, however, might have a negative PIB scan.

Diseases such as autoimmune reactions may produce a perivascular disease or a vasculitis, which, in the acute period, may course with slight hypermetabolism, but in the chronic period, produce hypometabolism. Given that the most sensitive part of the brain is the boundary of blood supply between cerebral arteries, the temporoparietal and the frontotemporal regions will be affected. In a more advanced stadium the patient may have symptoms of AD and the FDG may indicate a pattern similar to the one found in this disease.

The MCI patients classified as converters had amyloid depositions, but the FDG uptake was similar to the controls. The possibilities are that they had increased glucose uptake in the beginning of the amyloid depositions that normalized later; however, it is possible that amyloid depositions do not increase the glucose uptake because they occur slowly under years of progress as a silent, devastating chronic deterioration.

More studies are needed to either confirm or question these interpretations. Based on our findings we suggest that diseases with cognitive impairment causing hypermetabolism in the brain and without amyloid depositions are of a more acute nature. These diseases affect the corridors between arteria cerebri anterior media and posterior and are possibly caused by an autoimmune or inflammatory base. The symptoms may be reverted spontaneously or by treatment.

**Figure 2:**
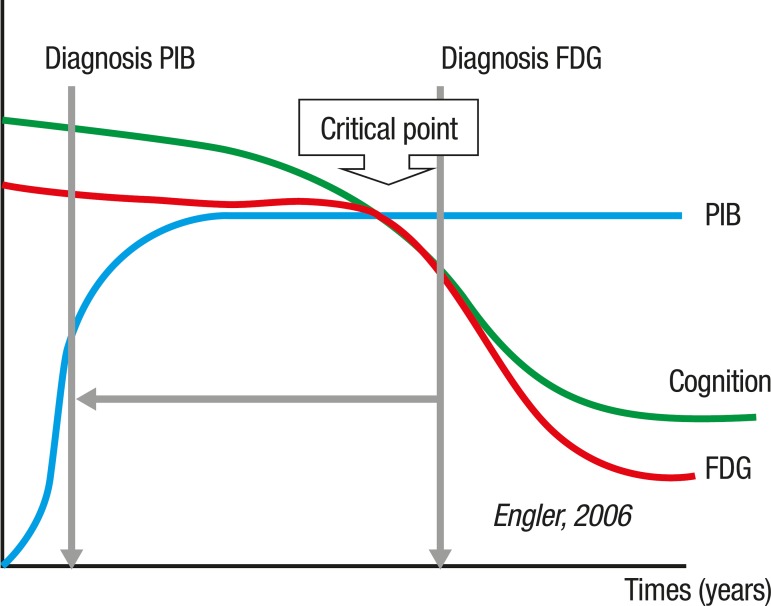
The amyloid deposition seems to begin early in the course of the disease, eventually reaching a "plateau". Glucose uptake is not affected during this period.

AD probably courses with normal metabolism until the damage, induced by the accumulation of plaques and tangles, reaches the critical point in which the neurons can no longer compensate the dysfunction and the symptoms appear (Figure 2).

There is possibly a critical point in which the capacity of the brain to compensate damage is overcome, with the consequence that symptoms appear. Cognitive impairment follows the decrease in glucose uptake but not the increase of amyloid deposition. FDG is a useful tracer to confirm the diagnosis of AD, but often when damage of brain tissue is pronounced. PIB may detect the presence of the protein before the neurodegenerative changes cause symptoms because the amyloid deposition occurs early in the course of the disease.

## PIB AND DOPA

Amyloid-containing deposits are a neuropathological feature in a wide range of dementias and movement disorders.

In a previous study^28^ we concluded that the normal PIB results in PD do not exclude the possibility that PIB binds to Lewy bodies, Lewy neurites or Amyloid-beta in PD.

Maetzler et al. examined ten patients with Parkinson's disease with dementia (PDD).^29^ Only two PDD patients displayed increased PIB binding to cortical amyloid comparable to AD patients. The remaining eight patients showed control-like cortical findings, but elevated PIB binding in the pons and mesencephalon. It has been suggested that, in addition to nonspecific binding, PIB uptake in the brainstem may also reflect PDD-related amyloid.^29^

Another study demonstrated the presence of a high affinity binding site for benzothiazole derivatives, including [3H]-PIB, on alpha-synuclein (AS) filaments generated *in vitro.*^30^

However, no discernible interaction of [3H]-PIB was detected with amygdala sections from PD cases containing frequent AS-immunoreactive Lewy bodies. These findings suggest that the density and/or accessibility of AS binding sites *in vivo* is significantly less than those associated with amyloid-beta peptide lesions. Lewy bodies pathology is therefore unlikely to contribute significantly to the retention of PIB in PET imaging studies.^30^

## PIB AND [^15^O]H2O CBF

In previous studies we have used FDG images based on a 40-60-minute summation as a template to realign PIB images because they give a good anatomic subtract allowing the definition of different brain regions.^2,23,31-33^ An automatic procedure has been applied to transfer the set of regions of interest (ROI) from FDG images to PIB images.^34^

In the case of PIB, it is difficult to define the brain cortex in HCs in a late PIB summation (40-60 min) because the tracer is retained only in the white matter disturbing the automatic realignment of images.

In the first study performed in Uppsala,^33^ we found that an early summation of the PIB activity frames (6 min) gave images that could be compared with later images of the FDG summation.

The early PIB summation images were realigned to the late FDG images and the rest of the PIB activity frames (7-60 min) were "co-resliced" using the realigned early summation as template.

In this way the ROIs drawn in the late FDG activity image could be transferred automatically to the later (40-60 min) PIB summation.

The similarity of the FDG images with the images of the early PIB summation suggested a positive relationship between the cumulative uptake of PIB in a short time interval following bolus administration and the cerebral blood flow (CBF) measured [^15^O]H2O.

A kinetic model with one reversible and one irreversible tissue compartment and three rate constants was used to investigate the PIB net accumulation (Kacc) and unidirectional influx (K1) across the blood brain barrier (BBB) in HC and AD-patients.^35^

This is in contrast to previous studies in which measures of PIB retention were obtained from reversible kinetic models.^36,37^ The blood input function was determined with arterial sampling in 4 HC-subjects and 4 AD-patients. Parametric maps of Kacc = K1k3/(k2+k3) and K1 were created using a linear algorithm.

Results were compared using the ratio between target uptake and a reference region in a late interval, in HC and AD patients from studies.^31,33^ The parameter Kacc and the late uptake ratio were found to have similar regional distributions.

The rate constant K1 for PIB was found to be comparatively larger, demonstrating high extraction of PIB into the brain tissue and indicating that this parameter might reflect CBF.

The possibility to replace K1 by a simple index of the PIB uptake in an early time interval following tracer administration was explored, but needs further validation.

**Figure 3:**
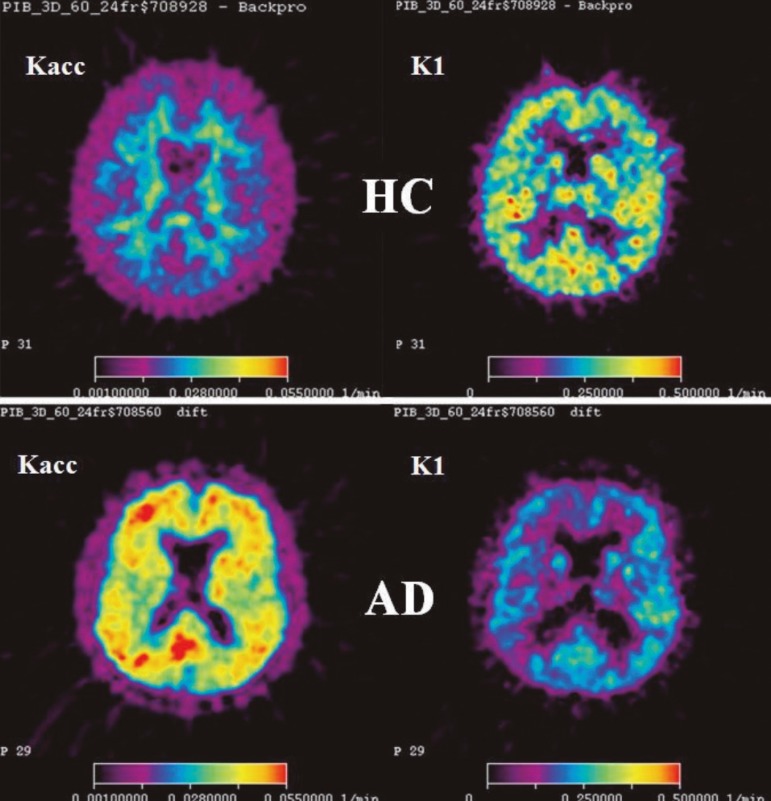
Parametric maps of the net accumulation rate constant Kacc (left) and the unidirectional influx rate constant K1 (right). Upper row: HC subject, lower row: AD-patient.

The regional K1 values were lower in AD patients than in HCs (Figure 3). Most AD patients had higher Kacc values than HCs in cortical areas, but some patients had values similar to HCs. However, these patients had lower K1 than HCs and did not differ from the other patients regarding this feature.

Furthermore, the effect on K1 and Kacc of CBF changes were investigated in an animal study using an anesthetized monkey and the following study design: PET scans were performed at baseline and 2 h later after increasing PaCO^2^ with the aid of respiratory control. At both experimental conditions 2 tracers [^15^O]H2O and PIB was performed within a short time interval. The unidirectional influx rate constant K1 of PIB was found to be a good index of CBF. The results indicate that the combined information of unidirectional influx and net accumulation of PIB might differentiate between groups of patients with an AD diagnosis.^35^

Many groups have supported this hypothesis, showing that PIB can be used as a functional and pathological tracer in AD.^38-40^ Recently Chen et al. compared the early dynamic PIB scans with [^15^O]H2O CBF studies, showing a high correlation with CBF in patients with AD.^41^ These findings confirm that PIB can also be used as a functional tracer in AD to explore areas of impaired CBF.
